# Inability to Catabolize Rhamnose by *Sinorhizobium meliloti* Rm1021 Affects Competition for Nodule Occupancy

**DOI:** 10.3390/microorganisms10040732

**Published:** 2022-03-29

**Authors:** Damien M. R. Rivers, Derek D. Kim, Ivan J. Oresnik

**Affiliations:** Department of Microbiology, University of Manitoba, Winnipeg Manitoba, MB R3T 2N2, Canada; damien.rivers@umanitoba.ca (D.M.R.R.); kimd3458@myumanitoba.ca (D.D.K.)

**Keywords:** *Sinorhizobium meliloti*, competition for nodule occupancy, rhamnose, metabolism

## Abstract

*Rhizobium leguminosarum* strains unable to grow on rhamnose as a sole carbon source are less competitive for nodule occupancy. To determine if the ability to use rhamnose as a sole carbon source affects competition for nodule occupancy in *Sinorhizobium meliloti*, Tn*5* mutants unable to use rhamnose as a sole carbon source were isolated. *S. meliloti* mutations affecting rhamnose utilization were found in two operons syntenous to those of *R. leguminosarum*. Although the *S. meliloti* Tn*5* mutants were complemented using an *R. leguminosarum* cosmid that contains the entire wild-type rhamnose catabolic locus, complementation did not occur if the cosmids carried Tn*5* insertions within the locus. Through a series of heterologous complementation experiments, enzyme assays, gene fusion, and transport experiments, we show that the *S. meliloti* regulator, RhaR, is dominant to its *R. leguminosarum* counterpart. In addition, the data support the hypothesis that the *R. leguminosarum* kinase is capable of directly phosphorylating rhamnose and rhamnulose, whereas the *S. meliloti* kinase does not possess rhamnose kinase activity. In nodule competition assays, *S. meliloti* mutants incapable of rhamnose transport were shown to be less competitive than the wild-type and had a decreased ability to bind plant roots in the presence of rhamnose. The data suggests that rhamnose catabolism is a general determinant in competition for nodule occupancy that spans across rhizobial species.

## 1. Introduction

Biological nitrogen fixation in rhizobium-legume symbiosis has been recognized as a key asset to advancing the forefront of sustainable agriculture, but the vast majority of commercially inoculated rhizobia fail to compete with indigenous rhizobial strains in the field [1,2]. As many of the indigenous rhizobial population may have poor nitrogen-fixing capability, the ability of the inoculum rhizobia to successfully occupy the nodule is crucial for establishing a productive symbiosis. In recent years, there have been numerous advances with respect to understanding the events during nodule development [3,4,5,6]. In comparison, the mechanisms and the determinants which allow a rhizobial strain to outcompete another during the host infection process remain relatively unexplored [2,7].

In *R. leguminosarum*, the capacity to metabolize a wide range of substrates has been correlated with the competitive fitness of strains with respect to nodule occupancy [7,8]. This may be the reason for the predominance of ABC-type carbohydrate transporters localized on the *S. meliloti* and *R. leguminosarum* megaplasmids [9,10]. In previous work, *R. leguminosarum* and *S. meliloti* mutants unable to catabolize specific carbohydrates were found to be severely outcompeted by their wild-type counterparts in nodule occupation. This phenotype has been observed in mutants incapable of catabolizing *myo*-inositol, glycerol, homoserine, erythritol, and rhamnose [11,12,13,14,15,16,17,18]. To date, only myo-inositol had been reported to affect the competitive phenotype in more than one species [11,17].

Rhamnose catabolism is one of the determinants which had been found to affect the ability of *R. leguminosarum* strains to compete for nodule occupancy. Rhamnose is present throughout the polysaccharide rhamnogalacturonan, a key component of the host cell wall [19]. It has been hypothesized that the degradation of rhamnogalacturonan may provide a source of rhamnose during infection thread development, granting a competitive edge to strains that are capable of utilizing this niche carbon source [15]. The locus required for rhamnose catabolism in *R. leguminosarum* was found to be plasmid encoded [20]. Tn*5* insertions were introduced to the locus, resulting in mutants that are unable to catabolize rhamnose. These mutants were determined to be severely impaired in its ability to compete for nodule occupancy [15]. Characterization of the locus revealed that it produces two transcripts: one consisting of an isomerase (RhaI) and a dehydrogenase/aldolase (RhaD), while the other transcript encoded a negative regulator (RhaR), an ABC-type transporter (RhaSTPQ), a mutarotase (RhaU) [21], followed by a sugar kinase (RhaK) [22]. In addition, on the basis of genetic data, it was suggested the rhamnose catabolic pathway in *R. leguminosarum* is initiated by RhaK [22].

Further characterization of *rhaK* revealed that the absence of RhaK abolished the transport of rhamnose into the cell [23]. The deletion of *rhaK* had no impact on the transcription or the translation of *rhaSTPQ*, suggesting that RhaK itself may be involved in the transport of rhamnose [23]. Linker scanning mutagenesis of *rhaK* from *R. leguminosarum* showed that its ability to affect transport can be genetically uncoupled from its kinase function [24]. Analysis of the predicted structure of RhaK and its associated alleles supported the possibility that it affects transport through a direct interaction with the ABC transporter. Investigation of the possible protein–protein interaction using the bacterial two-hybrid system revealed that the N- and C-terminal regions of RhaK could directly interact with RhaT, the ATP-binding protein of the transporter [25].

This work aims to address two hypotheses; (1) that the ability to catabolize rhamnose may be a determinant in competition for nodule occupancy for *S. meliloti*, and (2) to determine whether RhaK from *S. meliloti* has the same phenotypic characteristics as that from *R. leguminosarum*.

## 2. Materials and Methods

### 2.1. Bacterial Strains, Plasmids, and Media

The bacterial strains and plasmids used in this work are listed in Table 1. *S. meliloti* strains were routinely grown at 30 °C using Tryptone Yeast extract (TY) or Luria Bertani (LB) as a complex medium [26,27]. Both *R. leguminosarum* and *S. meliloti* strains were grown on VMM as a defined medium [28], which was modified as previously described [15]. Yeast extract mannitol (YEM) was used for strains grown for root attachment assays [28]. When required, antibiotics were used at the following concentrations: tetracycline (Tc), either 5 or 10 µg mL^−1^; neomycin (Nm), 200 µg mL^−1^; kanamycin (Kan), 50 µg mL^−1^; streptomycin (Sm), 200 µg mL^−1^; rifampicin (Rf), 100 µg mL^−1^; chloramphenicol (Cm), 20 µg mL^−1^; and gentamicin (Gm), 20 or 50 µg mL^−1^.

### 2.2. Genetic Techniques

Tn*5* mutagenesis of Rm1021 was carried out using pRK602 as previously described [34]. Putative mutants were purified three times before being tested for the correct phenotype. Strains that possessed the desired phenotype were subsequently transduced into Rm1021 to ensure that the Tn*5* and the marker were 100% linked by transduction [35]. Conjugations were executed as previously described [36].

Plasmids pDR32, pDR35, and pDR190 were constructed using the Gateway^TM^ compatible vector pCO37 [33]. This plasmid is a derivative of pRK7813 that has been modified such that it contains *attB1* and *attB2* sites that allow recombination from the ORFeome entry plasmid pMK2010. *S. meliloti* ORFeome clones [37] were recombined into pCO37 as previously described [38,39]. The ORFeome clones for *rhaI*, *rhaK*, and *rhaR* were used to construct pDR32, pDR35, and pDR190, respectively. The resulting constructs were completely nucleotide sequenced.

### 2.3. DNA Manipulations

Gel electrophoresis, restrictions, ligations, and PCR reactions were performed according to standard techniques [26]. Arbitrary PCR was employed to locate Tn*5* insertions in the genome and is described in a previous work [40]. Nucleotide sequencing was accomplished by cycle sequencing using a Big Dye, version 3.1 kit as recommended by the manufacturer and resolved using an ABI3130 sequencer.

### 2.4. Rhamnose Transport Assay

Transport assays were conducted as previously described [24]. Radioactive [^3^H] rhamnose (5 Ci/mmol) was purchased from American Radiolabeled Chemicals Ltd. (St. Louis, MO, USA). Transport assays were initiated by the addition of tritiated rhamnose to a final concentration of 2 µM. Aliquots of 0.5 mL were withdrawn at appropriate time points, and then rapidly filtered through a Millipore 0.45 µm Hv filter on a Millipore sampling manifold. Filtered cells were immediately washed with 5 mL of defined salts medium, and the residual radioactivity on the filter was quantified using a liquid scintillation spectrophotometer (Beckman LS6500). Transport rates were linear over the first minute of the assay.

### 2.5. Enzyme Assays

Preparation of *R. leguminosarum* and *S. meliloti* cell-free lysates were performed as previously described [41]. L-rhamnose isomerase activities were determined by measuring the formation of rhamnulose (ketose formation) as described in the cysteine-carbazole method [42,43]. Sugar kinase assays were carried out as described by [44], and modified to use rhamnose as a substrate [24].

### 2.6. β-Galactosidase Assays

*S. meliloti* cultures containing the transcriptional fusions were first grown overnight in either TY or LB broth and subsequently sub-cultured in defined medium containing pertinent carbon sources. Cultures used for these assays were at approximately 0.5 OD_600_. Assays were carried out essentially as described [45], except the assay times were based on the known activity of the fusions [22].

### 2.7. Plant Assays

Plant symbiotic assays were performed using alfalfa (*Medicago sativa* cv. Rangelander) as previously described [46]. Competition for nodule occupancy experiments were carried out as previously described [47]. Briefly, an overnight culture of *S. meliloti* in LB was diluted 1/100 in sterile distilled water and 10 mL of the dilution was used to inoculate alfalfa seedlings in a Leonard jar assembly, to approximately 10^5^ cfu/seedling. The ratio of the strains in the inoculum was determined by spread plating and screening an appropriate dilution of the inoculant. The competition phenotype was analyzed by comparing the ratio of the initial inoculum to the proportion of nodules occupied by each strain [47]. Statistical significance was determined using a Student’s *t* test.

Nodulation kinetics assays were carried out as previously described [48,49]. A total of 5 biological replicates, each containing approximately 10 plants, were used for each strain. Each seedling was inoculated with 100 μL of an overnight culture that was grown in LB and diluted 100-fold in sterile distilled water. Nodule formation was scored daily for 19 days.

To carry out root attachment assays, strains were grown overnight in YEM and diluted in Jensen’s medium. Seeds were surface sterilized and germinated on water agar [46]. Ten seedlings with an emerging root of approximately 1 cm were placed into a Petri plate containing 25 mL Jensen’s medium. Bacterial strains were then added to a titer of approximately 10^6^ cfu/mL. The seedlings and bacteria were incubated at room temperature for 90 min with gentle swirling (40 rpm). When used, glucose or rhamnose were added to a final concentration of 15 mM. Following incubation, each seedling had its cotyledon removed; the root was placed in a microfuge tube and washed to remove unbound bacteria. Each seedling was washed ten times, each wash consisted of 800 µL 0.85% saline. The seedling was finally resuspended into 200 μL saline and ground in the microfuge tube with a pestle. One hundred microlitres of this slurry was plated onto LB agar containing appropriate antibiotics to determine the total number of bacteria adhering to each root segment.

## 3. Results

### 3.1. Identification of a Rhamnose Catabolic Operon in S. meliloti

One thousand Tn*5* mutants from each of 10 independent mutagenesis experiments were screened for their inability to grow on rhamnose as a sole carbon source to identify mutations that can result in the loss of rhamnose catabolism in *S. meliloti*. Mutants possessing this phenotype were purified, retested, and then transduced into the wild-type to show that the transposon and the phenotype were 100% transducible (typically 50–100 colonies screened). Ten mutants were isolated from this endeavor (Table 1). In addition, a *tpiA* mutant was also isolated on the basis of a slow growth phenotype and has been previously reported [34].

**Figure 1:**
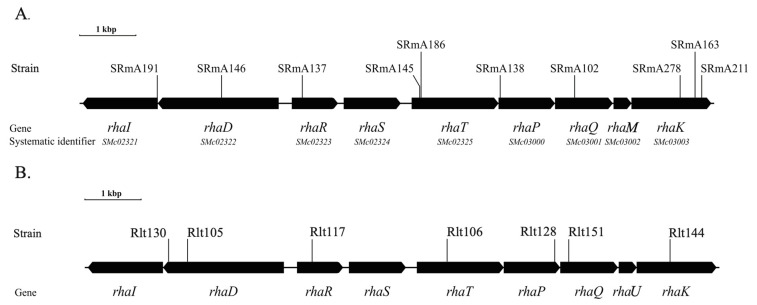
The rhamnose catabolic regions in *S. meliloti* strain Rm1021 (**A**), and *R. leguminosarum* bv. *trifolii* strain Rlt100 (**B**). Solid arrows represent the genes and the direction of transcription at each locus. Vertical lines represent sites of Tn*5* or Tn*5*-B20 insertion. Strain names corresponding to specific alleles are given above the genes, while gene names are given below the genes. In the case of *S. meliloti*, the systematic identifier numbers are also shown.

The site of the Tn*5* insertion in each of the mutants was determined by sequencing the product of an arbitrary PCR reaction that had used the genomic DNA from each of the mutants as template. Each of the inserts was localized to the genome of Rm1021 by using the generated sequence as a BLASTn query against the Rm1021 genome. The 10 mutations mapped to the locus were delineated as *SMc02321* and *SMc03003* (Figure 1). The genes at this locus have been annotated as *rha* based on sequence similarity and synteny to that of *R. leguminosarum* [22], as well as previous work which have identified genes in this region that have shown to affect rhamnose catabolism [10,33,34].

### 3.2. Complementation of S. meliloti Rhamnose Mutants with R. leguminosarum Rhamnose Catabolic Genes

Complementation cloning experiments were conducted to characterize the putative *rha* locus in *S. meliloti*. Attempts to isolate a complementing cosmid from two independent cosmid banks carrying DNA from Rm1021 were repeatedly unsuccessful. Subsequent screening of these pooled banks using PCR primers complementary to a region in *rhaP* failed to generate a PCR product, suggesting that the rhamnose locus of *S. meliloti* is either poorly represented or completely absent from these cosmid banks.

Considering this, heterologous complementation experiments were performed to provide corroborating evidence that the *S. meliloti* locus possesses the same operon structure as its *R. leguminosarum* counterpart. Cosmids containing either the *R. leguminosarum* wild-type *rha* region (pW3A1) or variants carrying a transposon in either one of the two transcripts (*rhaD* or *rhaT;* pW3AR1 and pW3AR2, respectively) were mobilized into appropriate *S. meliloti* and *R. leguminosarum* mutants. Resulting transconjugants were tested for their ability to grow in defined medium with rhamnose as a sole carbon source. As previously shown, pW3A1 complemented both *R. leguminosarum* mutants Rlt105 and Rlt106 [15]. Rlt105 was complemented by pW3AR2 but not pW3AR1, while Rlt106 was complemented by pW3AR1 but not by pW3AR2. The *S. meliloti* mutants were complemented by the cosmid pW3A1, which contains the entire wild-type locus, but not by either pW3AR1 or pW3AR2.

### 3.3. Growth of S. meliloti rhaDI Mutants Are Not Inhibited on Rhamnose/Glycerol Media

In *R. leguminosarum*, it was found that strains carrying either a *rhaDI* or a *rhaI* mutation were incapable of growing on defined medium containing rhamnose and glycerol [22], and that this phenotype was dependent on *rhaK* [22,23]. This is believed to be due an accumulation of phosphorylated rhamnose intermediates [22]. In order to assess the similarity of the rhamnose catabolism pathway in *S. meliloti*, mutants incapable of utilizing rhamnose were tested for their ability to grow on defined medium containing rhamnose and/or glycerol (Table 2). It was found that, while the *R. leguminosarum* strains carrying a *rhaDI* mutation failed to grow on defined medium containing rhamnose and glycerol, the *S. meliloti* mutants exhibited robust growth on either medium (Table 2).

### 3.4. S. meliloti rhaK (rhaK_Sm_) and rhaI (rhaI_Sm_) Can Complement R. leguminosarum Mutants

To resolve possible differences in the rhamnose metabolism, we wanted to determine whether the terminal genes in both operons (*rhaI* and *rhaK*) are capable of heterologously complementing the corresponding mutations in each species. Each of the genes was cloned into a broad-host range vector for expression. The genes *rhaI* and *rhaK* from *S. meliloti* (denoted as *rhaI_Sm_* and *rhaK_Sm_*) were recombined from the *S. meliloti* ORFeome into pCO37, yielding pDR32 and pDR35, respectively. We had previously constructed pMR110, which contains *rhaK* from *R. leguminosarum* (*rhaK_Rl_*) [23]. Although we were able to isolate multiple independent *rhaI* clones from *R. leguminosarum* (*rhaI_Rl_)* and verify them via nucleotide sequencing, the constructs were unable to complement *R. leguminosarum rhaI* mutations. The reason for their inability to complement was not pursued further. These constructs were subsequently conjugated into representative strains of *R. leguminosarum* and *S. meliloti* carrying *rhaK* and *rhaI* mutations. The results show that both *rhaK_Rl_* and *rhaK_Sm_* complemented *rhaK* mutations in both species. Similarly, *rhaI_Sm_* was able to complement *rhaI* mutations in both species (Table 3).

### 3.5. RhaK_Sm_ Does Not Possess Measurable Rhamnose Kinase Activity

In *E. coli*, the rhamnose catabolic pathway proceeds through the isomerization of rhamnose into a keto-sugar, followed by phosphorylation, and then finally undergoing an aldolase reaction to yield two three-carbon sugars; lactaldehyde and di-hydroxy-acetone phosphate [42,50,51,52]. In *R. leguminosarum*, it has been shown that RhaK has rhamnose kinase activity [21,23,24].

As the heterologous complementation experiments with *rhaI* and *rhaK* suggested that these genes were orthologous, we were unable to explain the lack of a conditional inability of *S. meliloti rhaDI* mutants to grow on defined medium containing glycerol and rhamnose. Our aim was to determine whether RhaK*_Sm_* and RhaI*_Sm_* possess the same biochemical activity as their *R. leguminosarum* counterparts.

The results show that cleared extracts from both *S. meliloti* and *R. leguminosarum* had inducible rhamnose isomerase activity (Table 4). These activities were absent in both strains carrying *rhaI* mutations, and restored with the introduction of *rhaI_Sm_* on a plasmid (Table 4). When the extracts were assayed for rhamnose kinase activity, it was found that Rlt100 had an inducible rhamnose kinase activity that was dependent upon the presence of *rhaK_Rl_*. Contrary to this, rhamnose kinase activity was not detected in Rm1021 (Table 4). This activity was observed in *S. meliloti* transconjugants that carried *rhaK_Rl_*, but not by transconjugants that carried *rhaK_Sm_* (Table 4). Moreover, *R. leguminosarum* mutant Rlt144 complemented by *rhaK_Sm_* for growth using rhamnose failed to show any levels of rhamnose kinase activity above basal rates (Table 4).

### 3.6. The Rhamnose Transporter RhaSTPQ Is Required for Growth on Rhamnose

**Figure 2:**
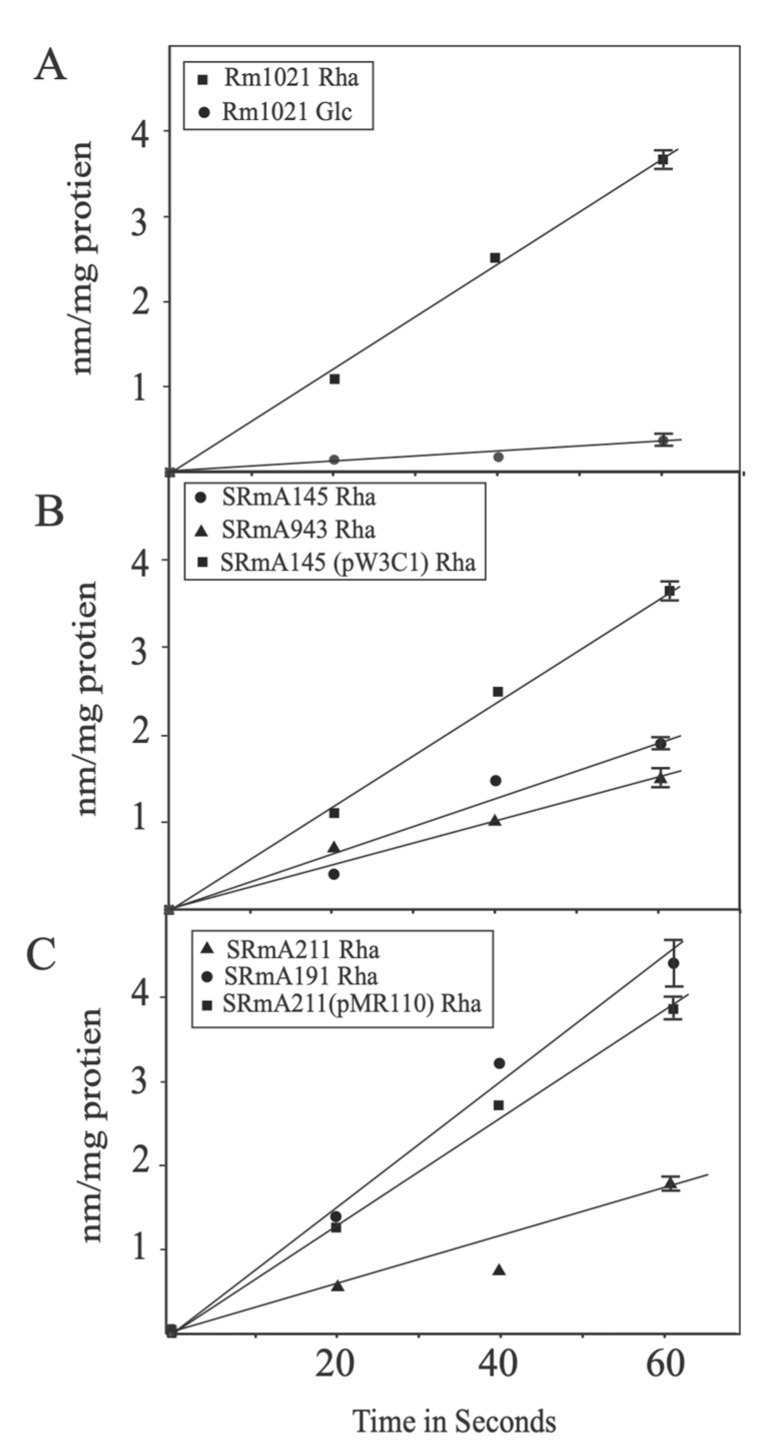
Rhamnose transport assays. Strains were grown to mid-log phase in defined medium containing either glucose/glycerol (Glc) or rhamnose/glycerol (Rha) as indicated. Transport rates were determined using (^3^H) rhamnose as described in materials and methods. (**A**) Rhamnose uptake between Rm1021 grown on glucose or rhamnose. (**B**) Comparison of rhamnose uptake of induced ABC transporter mutants and complemented transporter mutant. (**C**) Rhamnose transport by induced kinase and isomerase mutants as well as a complemented kinase mutant. Symbols for strains are indicated on the figure inset. The data shown represent averages of biological three replicates. Error bars represent standard deviation. Where not seen, error bars are smaller than the representative symbols. Data shown in all three panels were carried out at the same time. For clarity, the data is presented in three separate panels.

To provide direct evidence that the ABC-type transporter consisting of RhaSTPQ was responsible for rhamnose uptake, transport assays were carried out using uniformly labelled [^3^H] rhamnose. We were able to determine that *S. meliloti* grown on defined medium with glucose had negligible rates of rhamnose uptake, whereas cells grown on rhamnose as a sole carbon source showed significant rates of rhamnose uptake (Figure 2A). The typical transport rate of Rm1021 in our growth conditions was approximately 3.5 nmol/min/mg protein, while the typical rate measured for *R. leguminosarum* is greater than twice this value (10 nmol/min/mg protein) [24].

Performing this assay on the *rhaT* mutant SRmA145 resulted in considerably lower transport rates than the rhamnose induced wild-type cells, but substantially greater than those observed in glucose-grown cells (Figure 2B). The polar nature of the insertion mutation in SRmA145 affected *rhaK*, which has been linked to rhamnose transport in *R. leguminosarum* [24]. Taken together, this raised the possibility that the inability to grow may not be solely attributed to the lack of *rhaTPQ*. To address this, a deletion of *rhaP* was constructed utilizing the *S. meliloti* ORFeome, such that the downstream genes could still be transcribed [37,38]. The resultant strain was verified by nucleotide sequencing of the region and was named SRmA943. This mutant was unable to grow with rhamnose as a sole carbon source. Transport assays of the strain carrying the *rhaP* deletion using radio-labelled rhamnose yielded rates that were not statistically different from SRmA145 (Figure 2B). We note that when SRmA943 was incubated for an extended period of time on defined medium with rhamnose as a sole carbon source (greater than 10 days), very weak growth was observed.

Rhamnose uptake was assayed in SRmA211, a *rhaK* Tn*5* insertion mutant, to determine if RhaK affects transport in *S. meliloti* like in *R. leguminosarum*. The results show that SRmA211 had rates that were comparable to the *rhaT*::Tn*5* mutant SRmA145, as well as the *rhaP* deletion mutant SRmA943 (Figure 2B). Introduction of either *rhaK_Rl_* or *rhaK_Sm_* on a plasmid restored the ability to grow on rhamnose as a sole carbon source as well as transport rhamnose at rates comparable to Rm1021 (Figure 2C). This suggests that RhaK_Sm_ and RhaK_Rl_ both affect rhamnose transport. Transport of rhamnose was unaffected by a Tn5 insertion in rhaI in strain SRmA 191 (Figure 2C).

### 3.7. Cosmids Carrying the R. leguminosarum Rhamnose Locus Show Reduced Expression in Rm1021

The *R. leguminosarum* rhamnose locus has been shown to be negatively regulated by RhaR [22]. Although *rhaRSTPQUK* were empirically shown to be a single transcriptional unit, the transcription of *rhaRS* occurred under non-inducing conditions, while the transcription of the full operon occurs under inducing conditions [22]. Full induction of this operon was also independent of the presence of the ABC transporter or *rhaK* [22,23]. As both *rhaI_Rl_* and *rhaK_Rl_* have been shown to complement its corresponding *S. meliloti* mutants, it is unlikely that the failure to complement mutations in the *S. meliloti* rhamnose region using cosmids carrying transposon insertions in the *R. leguminosarum* rhamnose catabolism locus is caused by biochemical differences in the encoded enzymes between the two species. This led to the hypothesis that this inability stems from regulatory differences, suggesting that RhaR*_Sm_* acts in a dominant fashion to RhaR*_Rl_*.

To test this, *R. leguminosarum* cosmid-borne transcriptional fusions located in each of the transcripts were introduced into both Rm1021 as well as Rlt100. The data show that the *rhaP36* and the *rhaD1* fusions were induced by greater than 6 and 5-fold in Rlt100, respectively (Table 5). These same fusions were either uninduced or marginally induced in Rm1021 (Table 5). When the *R. leguminosarum rhaD1* fusion was introduced into an *S. meliloti rhaR* background, this fusion was capable of induction by rhamnose and the basal expression under non-inducing conditions was higher (Table 5).

To determine if *rhaR_Sm_* acted as a dominant negative allele, pDR190 (*rhaR_Sm_* on a broad-host vector) was introduced into the *R. leguminosarum* strains Rlt128 and Rlt151 that had chromosomal transcriptional fusions in the r*haP* and *rhaQ*, respectively. As a control, pMR53, which carries the *R. leguminosarum rhaR*, was also introduced into these strains. Whereas the introduction of pMR53 did not affect the induction of these fusions in the presence of rhamnose, the introduction of pDR190 did reduce the expression of the chromosomal transcriptional fusions alleles, corroborating the plasmid fusion data (Table 5).

### 3.8. S. meliloti Rhamnose Mutants Are Less Competitive for Nodule Occupancy on Alfalfa

**Figure 3:**
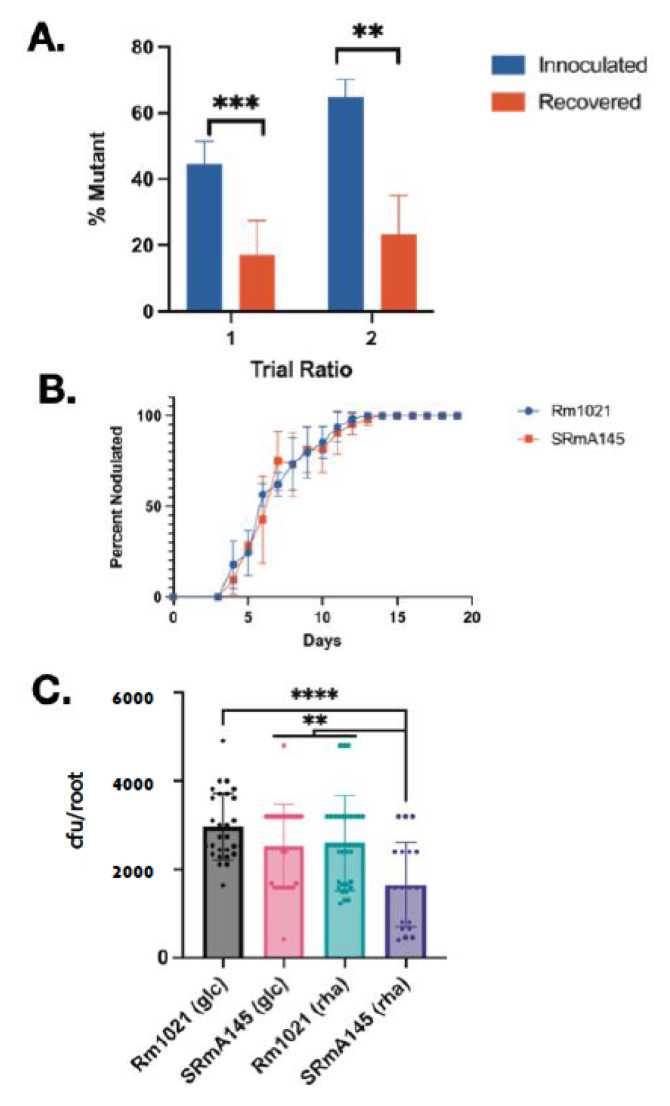
Experimental characterization of plant interaction phenotypes. (**A**) Competition for nodule occupancy assays between Rm1021 (wild-type) and the rhamnose catabolic mutant SRmA145 (*rhaT*::Tn*5*). SRmA145 was inoculated with the wild-type onto alfalfa plants at two ratios. Blue bars represent the percentage of mutant in the inocula; red bars represent the percentage of nodules occupied by the mutant strain as determined by bacterial isolation from nodules. Ratio 1, *n* = 6, *p* < 0.0003; Ratio 2, *n* = 5, *p* < 0.009). Significance was determined using an unpaired *t*-test. (**B**) Nodulation kinetics assays were carried out as described in materials and methods. The data is presented as the mean and error bars represent standard deviation. (**C**) Root attachment assays from single strain inoculations (10^5^ cfu/seedling) as described in materials and methods. Solid bars represent the mean. Error bars represent the standard deviation. Individual data points are represented as solid points. Significance was determined using a one way ANOVA and post hoc unpaired t-test. **** *p* < 0.0001; *** *p*< 0.001; and ** *p* < 0.01.

*R. leguminosarum* bv. *Trifolii* mutants unable to catabolize rhamnose were previously determined to be severely defective in their ability to compete for nodule occupancy [15]. To determine if the inability to use rhamnose would also affect competition for nodule occupancy in *S. meliloti*, a mixed inoculum of SRmA145 and Rm1021 was inoculated onto alfalfa. Two inoculum ratios were used, consisting of approximately an equivalent proportion of mutant:wild-type (45 ± 7%) and a 2:1 ratio of mutant:wild-type (65 ± 5%). The resulting nodules were harvested after 28–35 days. In each case, the nodule occupancy was significantly lower than the inoculum ratio (Figure 3A).

Very little work has been focused on determining why *R. leguminosarum* strains unable to utilize rhamnose are not competitive for nodule occupancy. Work had focused on the novel aspects of the RhaK and its ability to affect transport [21,23,24,25]. To address the hypothesis that rhamnose plays a role during nodule initiation, nodulation kinetics experiments were carried out. The results show that the strain carrying the mutation initiated nodules at the same rate as the wild-type (Figure 3B), suggesting that competition for nodule occupancy must occur at an early stage of interaction. To test this, the mutant strain and the wild-type were tested for their ability to adhere to germinating seedlings. The results of these experiments showed that, whereas there was no significant difference between the wild-type and the mutant strain if they were incubated with seedlings in the presence of glucose, the mutant showed a significant difference to the wild-type in the presence of both glucose and rhamnose, as well as significant difference with its ability to adhere depending on whether it was incubated in glucose or rhamnose (Figure 3C).

## 4. Discussion

There are a number of similarities and differences between the *R. leguminosarum* and *S. meliloti* with respect to the protein identity of the initial two proteins responsible for the metabolism of rhamnose. The isomerases are predicted to have 78% identity and 90% similarity, whereas the kinases are predicted have 55% identity and 67% similarity. In this work, we have shown that the metabolism of rhamnose in *R. leguminosarum* and *S. meliloti* consists of the initial isomerization of rhamnose into rhamnulose by RhaI before being phosphorylated into rhamnulose phosphate by RhaK. This conclusion is based on biochemical assays for rhamnose isomerase and kinase activity in both organisms, as well as heterologous complementation experiments (Table 3 and Table 4). This pathway has been observed in other organisms [53,54]. Thus, the primary function of RhaK in both species is suggested to be a rhamnulose kinase, and the rhamnose kinase activity reported in *R. leguminosarum* is unlikely to be required for catabolism. It is unclear why RhaK*_Rl_* possesses the ability to directly phosphorylate rhamnose, or the physiological function of its product, rhamnose phosphate We note that metabolic by-products have been found to play a regulatory role [55].

Unlike the *R. leguminosarum* rhamnose transporter mutants, the transporter mutants *of S. meliloti* have exhibited a transport rate greater than the uninduced wild-type strain (Figure 2). Although this transport rate was markedly greater than the basal rates, it failed to result in growth on defined medium supplemented with rhamnose as a sole carbon. While the physiological implications of this residual transport rate are unclear, we note that extended incubation of SRmA943 (∆*rhaP*) on rhamnose as a sole carbon source exhibited extremely weak growth, whereas SRmA145 (*rhaT*::Tn*5*) failed to grow.

There have been numerous attempts to complement *S. meliloti rha* mutations using cosmids carrying the *rha* locus originated from *R. leguminosarum*. Cosmid-borne fusion experiments reported significant levels of expression in *R. leguminosarum* while exhibiting substantially reduced levels in *S. meliloti* (Table 5). The reason for this has been attributed to the negative dominance of RhaR*_Sm_* over RhaR*_Rl_*. RhaR is predicted to be a DeoR-type negative regulator and is consistent with its characterization in a previous work [22]. The alignment of the RhaR amino acid sequences between the two species show 68% identity, with their DNA-binding helix-turn-helix motifs showing a four-amino-acid variance across 62 amino acids. Although clear differences between the two RhaR proteins exist, the mechanism of the negative dominance has yet to be elucidated.

The main objective of this work is to determine if rhamnose catabolism plays a role in competition for nodule occupancy in *S. meliloti* as it does in *R. leguminosarum*. Competition for nodule occupancy assays show that *S. meliloti* rhamnose mutants are compromised in their ability to compete against an isogenic wild-type (Figure 3A). In addition, they are less capable of adhering to germinating seedlings (Figure 3C). A mariner-based transposon insertion sequencing study recently identified that insertions in *rhaD* (pRL110415) resulted in an impaired fitness with respect to survival in the nodule or bacteroid, whereas mutations in the rhamnose transporter (pRL110413-pRL110410) did not show the same phenotype [56]. Although it was suggested this might be due to a second rhamnose transporter that might be present in the Rlv3841 genome, based on the ability of RhaK*_Rl_* to directly phosphorylate rhamnose [23], the close phylogenetic relation of RhaK*_Rl_* with the Rlv3841 homologue (pRL110408) [25] and that insertions within pRL110413-pRL110410 would be polar on pRL110408; an alternative hypothesis could be that rhamnose phosphate is being generated in strains carrying *rhaD* mutations which cannot be further metabolized and leads to impaired nodule and bacteroid fitness.

In both *R. leguminosarum* strain Rlt100 as well as *S. meliloti*, it was shown that strains carrying mutations in their transporters were unable to compete for nodule occupancy ([15], Figure 3A), suggesting that rhamnose catabolism affects competition for nodule occupancy across multiple rhizobial species. Based on our data, it is possible rhamnose is available during the host colonization process, allowing strains capable of its catabolism a competitive edge. Supporting this hypothesis, small amounts of rhamnose have been measured in the root exudates in other legumes [57]. Alternately, it is also possible that that this inability to compete for nodule occupancy is not directly linked to the ability to use the rhamnose as a carbon source, but due to its presence which cannot be reduced if it is not metabolized. The exact mechanism of how rhamnose affects competition for nodule occupancy is the focus of future work.

## Figures and Tables

**Table 1:** Strains and plasmids.

Stain or Plasmid	Genotype or Phenotype	Reference or Source
Strains		
*S. meliloti*		
Rm1021	SU47 *str-21*; Sm^r^	[29]
SRmA102	Rm1021, *rhaQ*::Tn*5*, (Nm^r^)	(This work)
SrmA137	Rm1021, *rhaR*::Tn*5*, (Nm^r^)	(This work)
SrmA138	Rm1021, *rhaP*::Tn*5*, (Nm^r^)	(This work)
SrmA145	Rm1021, *rhaT*::Tn*5*, (Nm^r^)	(This work)
SrmA146	Rm1021, *rhaD*::Tn*5*, (Nm^r^)	(This work)
SrmA163	Rm1021, *rhaK*::Tn*5*, (Nm^r^)	(This work)
SrmA186	Rm1021, *rhaT*::Tn*5*, (Nm^r^)	(This work)
SrmA191	Rm1021, *rhaI*::Tn*5*, (Nm^r^)	(This work)
SrmA211	Rm1021, *rhaK*::Tn*5*, (Nm^r^)	(This work)
SrmA278	Rm1021, *rhaK*::Tn*5*, (Nm^r^)	(This work)
SrmA943	Rm1021, Δ*rhaP*	(This work)
*R. leguminosarum*		
Rlt100	W14-2, wild-type (Sm^r^)	[15]
Rlt105	Rlt100 *rhaD1*::Tn*5*-B20, (Nm^r^)	[15]
Rlt106	Rlt100 *rhaT2*::Tn*5*-B20, (Nm^r^)	[15]
Rlt117	Rlt100 *rhaR25*::Tn*5*-B20, (Nm^r^)	[22]
Rlt128	Rlt100 *rhaP36*::Tn*5*-B20, (Nm^r^)	[22]
Rlt130	Rlt100 *rhaI39*::Tn*5*-B20, (Nm^r^)	[22]
Rlt144	Rlt100 *rhaK50*::Tn*5*-B20, (Nm^r^)	[22]
Rlt151	Rlt100 *rhaQ38*::Tn*5*-B20, (Nm^r^)	[22]
*E. coli*		
DH5α	*endA hsdR17 supE44 thi-1 recA1 gyrA96 relA1 (argF-lacZYA) U169 80 dlacZ M15*	[30]
MT616	MT607 (pRK600)	[31]
Plasmids		
pRK7813	Broad host range vector, Tc^r^	[32]
pCO37	pRK7813 containing *attB* sites; Gateway-compatible destination vector	[33]
pRK600	pRK2013 *npt*::Tn*9*, Cm^r^	[31]
pW3A	*R. leguminosarum* rhamnose locus in pRK7813	[15]
pW3C1	*R. leguminosarum* rhamnose locus in pRK7813	[15]
pW3AR1	pW3A1, *rhaD1:*:Tn*5*-B20	[15]
pW3AR2	pW3A1, *rhaT2*::Tn*5*-B20	[15]
pMR84	pW3C1, *rhaP36*::Tn*5*-B20	[22]
pMR110	*R. leguminosarum rhaK^+^* in pRK7813	[23]
pMR53	*R. leguminosarum rhaR^+^* in pRK7813	[22]
pDR32	*S. meliloti rhaI*^+^ in pCO37	(This Work)
pDR35	*S. meliloti rhaK*^+^ in pCO37	(This Work)
pDR190	*S. meliloti rhaR*^+^ in pCO37	(This Work)

Abbreviations for antibiotics are as follows: Cm, chloramphenicol; Nm, neomycin; Sm, streptomycin; and Tc, tetracycline.

**Table 2:** *S. meliloti* displays different conditional growth phenotypes than *R. leguminosarum*.

Strain.	Relevant Characteristics Chromosomal (Plasmid)	Glyc	Rham	Rham/Glyc
Rlt100	wild-type	+	+	+
Rlt144	*rhaK*	+	−	+
Rlt105	*rhaDI*	+	−	−
Rm1021	wild-type	+	+	+
SRmA211	*rhaK*	+	−	+
SRmA146	*rhaDI*	+	−	+
SRmA191	*rhaI*	+	−	+
SRmA191 (pDR32)	*rhaI* (*rhaI^+^*)	+	+	+
SRmA146 (pDR32)	*rhaDI* (*rhaI^+^*)	+	−	±

+ indicates ability to grow on VMM minimal medium plus indicated carbon source; − indicates inability to grow on VMM minimal medium plus indicated carbon source; ± indicates weak growth on VMM minimal medium plus indicated carbon source.

**Table 3:** Plasmid encoded *rhaK* and *rhaI* can heterologously complement corresponding mutations in both *S. meliloti* and *R. leguminosarum*.

Strain	Relevant Characteristics	pDR32 (*rhaI*_Sm_) ^a^	pDR35 (*rhaK*_Sm_)	pMR110 (*rhaK*_Rl_)
Rlt100	*R. leguminosarum*, wild-type	+	+	+
Rlt144	*R. leguminosarum*, *rhaK^−^*	−	+	+
Rlt130	*R. leguminosarum*, *rhaI^−^*	+	−	−
Rm1021	*S. meliloti*, wild-type	+	+	+
SRmA211	*S. meliloti*, *rhaK*^−^	−	+	+
SRmA191	*S. meliloti*, *rhaI*^−^	+	−	−

Growth on VMM was scored as indicated; +, growth comparable to wild-type; −, no growth. ^a^ Subscript *_Sm_* or *_Rl_* following gene designation refers to the origin of the gene as *S. meliloti* or *R. leguminosarum,* respectively.

**Table 4:** Kinase and isomerase activity *of R. leguminosarum* and *S. meliloti* strains.

		Kinase Activity ^a^		Isomerase Activity ^b^	
Strain	Relevant Characteristics	Glc	Rha ^c^	Glc	Rha ^c^
Rlt100	*R. leguminosarum*, wild-type	23 ± 3	211 ± 3	48 ± 4	180 ± 6
Rlt144	Rlt100, *rhaK50*	44 ± 6	50 ± 27	^e^	
Rlt144 (pMR110)	Rlt100, *rhaK50* (*rhaK^+^_Rl_*)		640 ± 40 ^d^		
Rlt144 (pDR35)	Rlt100, *rhaK50* (*rhaK^+^_Sm_*)		50 ± 15 ^d^		
Rlt130	Rlt100, *rhaI*			ND ^f^	43 ± 9
Rlt130 (pDR32)	Rlt100, *rhaI* (*rhaI^+^_Sm_*)				269 ± 8 ^d^
Rm1021	*S. meliloti,* wild-type	18 ± 9	19 ± 6	65 ± 7	227 ± 32
SRmA211	Rm1021, *rhaK*	13 ± 3	19 ± 3		
SRmA211 (pMR110)	Rm1021, *rhaK* (*rhaK^+^_Rl_*)		383 ± 18 ^d^		
SRmA211 (pDR35)	Rm1021, *rhaK* (*rhaK^+^_Sm_*)		33 ± 8 ^d^		
SRmA191	Rm1021, *rhaI12*			ND	12 ± 1
SRmA191(pDR32)	Rm1021, *rhaI12* (*rhaI^+^_Sm_*)			ND	152 ± 31

Abbreviations: Glc, glucose; Rha, rhamnose. ^a^ Data presented as µmoles/min/mg protein. ^b^ Data presented as milliunits/min/ mg total protein. ^c^ Strains unable to grow on rhamnose as a sole carbon source were supplemented with glycerol in addition to rhamnose. ^d^ Genes encoded on pRK7813 are constitutively expressed from a *plac* promoter. Previous experiments confirmed that similar results are obtained when measuring activities in inducing and non-inducing conditions. Therefore, activities were only measured in one condition. ^e^ Assays where a value is not presented were not determined. ^f^ Not detected.

**Table 5:** Induction of *R. leguminosarum* cosmid-borne fusions in *R. leguminosarum* or *S. meliloti*.

Strain	Relevant Characteristics	Glc	Rha	Induction ^a^
Rlt100	*R. leguminosarum,* wild-type	30 ± 8	77 ± 12	-
Rm1021	*S. meliloti,* wild-type	7 ± 3	11 ± 4	-
Rlt128	Rlt100 *rhaP36*::Tn*5*-B20	74 ± 18	1336 ± 16	18.1
Rlt151	Rlt100 *rhaQ38*::Tn*5*-B20	46 ± 4	880 ± 94	19.1
SRmA137	Rm1021 *rhaR*::Tn*5*	6 ± 2	9 ± 4	-
Rlt100 (pMR84)	Rlt100 (*rhaP36*::Tn*5*-B20)	54 ± 3	362 ± 20	6.7
Rm1021 (pMR84)	Rm1021 (*rhaP36*::Tn*5*-B20)	122 ± 20	217 ± 20	1.8
Rlt100 (pW3CR1)	Rlt100 (*rhaD1*::Tn*5*-B20)	153 ± 10	832 ± 27	5.4
Rm1021 (pW3CR1)	Rm1021 (*rhaD1*::Tn*5*-B20)	207 ± 18	208 ± 34	1.0
SRmA137 (pW3CR1)	SrmA137 (*rhaD1*::Tn*5*-B20)	602 ± 31	1934 ± 18	3.2
Rlt128(pMR53)	Rlt128 (*rhaR_Rl_*)	75 ± 5	1591 ± 19	21.2
Rlt128(pDR190)	Rlt128 (*rhaR_Sm_*)	74± 5	202 ± 36	2.7
Rlt151(pMR53)	Rlt151(*rhaR_Rl_*)	56 ± 3	795 ± 109	14.2
Rlt51(pDR190)	Rlt151(*rhaR_Sm_*)	63 ± 2	192 ± 12	3.0

The values represent β-galactosidase activity expressed in Miller units following growth in defined media containing either glucose-glycerol (Glc) or rhamnose-glycerol (Rha) as carbon sources. Values are the means of three biological replicates and are presented as mean ± standard deviation. **^a^** Induction is expressed as the ratio of β-galactosidase activity of rhamnose values to glucose values. Inductions are only calculated for strains carrying *lacZ* fusions.

## Data Availability

Not applicable.
